# Heavy Menstrual Bleeding: Experiences and Learnings From Patient Survey

**DOI:** 10.1111/ajo.70143

**Published:** 2026-05-11

**Authors:** Mikaela Jacka, Madeleine Ward, Katrina Guerin, Russell Dalton

**Affiliations:** ^1^ Obstetrics & Gynaecology Illawarra Shoalhaven Local Health District Wollongong New South Wales Australia; ^2^ OGB Surfcoast, School of Medicine Deakin University Waurn Ponds Victoria Australia; ^3^ School of Medicine Deakin University Waurn Ponds Victoria Australia; ^4^ Obstetrics & Gynaecology Ballarat (OGB), School of Medicine Deakin University Wendouree Victoria Australia

**Keywords:** heavy menstrual bleeding, hysterectomy, treatment regret

## Abstract

**Background:**

Heavy menstrual bleeding (HMB) affects 1 in 4 women. It is often debilitating for those experiencing it, resulting in negative impacts on quality of life. Prompted by a national call for better insights into the management of HMB, the authors sought to collect patient feedback regarding access to HMB treatment alongside satisfaction with care, including treatment side effects, decision regret and the alignment of patient and clinician treatment goals.

**Aims:**

This study aimed to gather patient feedback on the management of HMB treatment access, satisfaction, side effects, decision regret and alignment of patient and clinician treatment goals.

**Methods:**

A retrospective recall survey was distributed to eligible participants at a single referral centre in regional Victoria, Australia. The survey was sent out to 1241 women who received treatment for heavy menstrual bleeding (HMB) between 01/01/2018 and 01/05/2023.

**Results:**

There were 150 respondents to the survey, with 84% reporting a wait time of less than 2 months to see a clinician. Satisfaction with symptom control was reported by 96.9% of hysterectomy patients, while 9.3% reported intolerable side effects and 7.7% expressed regret. Symptom control was less effective for oral hormones (39.5%), non‐oral hormones (62.5%) and Mirena (48.4%), which also had higher rates of intolerable side effects (47.2%, 62.5% and 42%) and regret (18%, 41.7% and 27.4%).

**Conclusion:**

The study underscores the need for personalised patient care in managing HMB, emphasising the importance of educating patients about treatment outcomes to support informed decision‐making.

## 1 Background

Heavy menstrual bleeding (HMB) is a common condition, affecting approximately one in four women over their lifetime [1, 2]. It is defined as excessive menstrual blood loss that interferes with physical, emotional, social, and material quality of life [2, 3, 4, 5]. HMB can cause debilitating symptoms such as fatigue, pain, and infertility [2, 4]. Abnormal uterine bleeding, including HMB, accounts for a substantial proportion of healthcare utilisation/expense, and lost productivity [6], representing a major healthcare burden.

Management of HMB includes both medical and surgical options. Medical treatments such as the oral contraceptive pill (OCP) and the levonorgestrel intrauterine device (LNG‐IUD), are commonly employed, but vary in efficacy. For instance, the LNG‐IUD is associated with a discontinuation rate of 23% within 36 months due to unpredictable bleeding profiles, despite its five‐year therapeutic potential [7]. A study by Cooper et al. [8] demonstrated that 59% of women using the OCP and 13.5% using the LNG‐IUD for HMB eventually progressed to surgical intervention within 2 years. A study by Beelen et al. [9] found that women who received the LNG‐IUS are more likely to need a subsequent additional treatment, when compared to those who had undergone endometrial ablation (EA) or endometrial resection (ER).

Surgical interventions like EA and ER are cost‐effective and offer definitive solutions but carry risks and long‐term implications [10, 11]. EA and ER are broadly classified as generation one or generation two, depending on the technique used. First generation techniques require a high degree of skill in hysteroscopy and include transcervical resection of the endometrium, rollerball ablation and laser ablation [12]. Second‐generation techniques avoid the need for hysteroscopy and include high temperature fluids within a balloon (Therachoice and Cavaterm), bipolar radiofrequency electrical energy (Novasure) and microwave energy (Microsulis) [12]. Studies have shown that 19% of patients who undergo EA/ER for HMB require a subsequent hysterectomy within 2 years [11], with 12%–25% feeling dissatisfied with EA/ER after two years due to recurrent HMB and/or cyclical pain [11]. A study by Campbell, Monaghan, and Parker [13] focussing specifically on EA with NovaSure with a treatment cohort size of 368, found that 87% were satisfied with the procedure (95% at 1 year), 59% experienced control of HMB (amenorrhea), and 7.6% subsequently required hysterectomy for their symptoms. Uterine length was found to be a predictive factor for success of EA, with those with a longer uterine cavity length being more likely to need subsequent treatment for HMB—based on these findings, EA with Novasure was deemed an effective treatment for HMB, although no data was reported on longer‐term follow‐up beyond one year [13].

A systematic review and meta‐analysis by Bergeron et al. [14] did not find any significant differences in the therapeutic effects, risks of requiring hysterectomy in the future, quality of life, or patient satisfaction when comparing either EA or ER with the LNG‐IUD, although this was somewhat dependent upon the age of the patient, with younger patients (< 42 years of age) found to be at higher risk of EA/ER failure and more side‐effects reported by those in the LNG‐IUD group. The use of first versus second generation techniques was not found to affect the results. These findings underscore the importance of individualised treatment planning to optimise outcomes.

Hysterectomy, while considered the only definitive treatment for HMB, has been subject to scrutiny due to its associated risks and rates of utilisation [15]. Australian hysterectomy rates are among the highest globally, second only to USA and Canada [16]. By age 60, 33% of Australian women have undergone the procedure [15, 16]. Regional variations in hysterectomy rates have been highlighted in reports, with some areas in regional Victoria demonstrating rates almost seven times higher than regions with the lowest rates [15]. These disparities have raised concerns about the overuse of hysterectomy in lieu of non‐surgical alternatives, as noted by The Australian Commission on Safety and Quality in Health Care (ACSQHC) [15, 17].

Globally, advancements in hysterectomy techniques and increased patient awareness have contributed to a rise in the number of hysterectomies performed for abnormal uterine bleeding [18]. However, this trend has also been attributed to inadequate access to less invasive treatments during earlier periods [6]. Despite its definitive nature, hysterectomy may be associated with chronic postoperative pain in a subset of patients, with studies reporting new or increased pain in 1%–15% of women within three months post‐hysterectomy [19, 20]. Factors such as preoperative depression, significant perioperative pain, and genetic susceptibility have been implicated in the development of post‐hysterectomy pain [19, 20].

Approximately 80% of patients undergoing hysterectomy for benign conditions have reportedly tried less invasive treatments beforehand [21]. Patient satisfaction post‐hysterectomy has been linked to the achievement of preoperative goals and preparedness for surgery [21]. Hysterectomy has demonstrated significant improvements in health‐related quality of life and high rates of patient satisfaction, particularly when aligned with patient expectations and appropriate indications [18].

In light of the ACSQHC findings [15] and the high hysterectomy rates in certain Victorian regions, a review of evidence‐based practices and patient satisfaction is warranted [22]. Given the primary goal of hysterectomy for HMB is to improve quality of life, patient‐reported symptom control remains a crucial measure of the procedure's effectiveness and safety.

## 2 Materials and Methods

### 2.1 Study Design

A retrospective recall survey was conducted among women treated for HMB at a single referral centre in regional Victoria, Australia.

### 2.2 Participants and Recruitment

The study clinic's medical database was used to recruit participants who sought care for the issue of heavy menstrual bleeding from 1st January 2018 to 1st May 2023. Eligible participants were invited to complete an anonymous Qualtrics patient follow‐up survey via an electronic link, with a reminder sent two weeks later.

### 2.3 Survey Design

Participants completed a survey designed by the study team, which included questions on referral pathway and diagnostics; treatments utilisation and outcomes; patient satisfaction, goals, and regret. Participants were asked to rank their level of agreement with statements by selecting one of ‘strongly agree’, ‘somewhat agree’, ‘neither agree nor disagree’ (neutral), ‘somewhat disagree’ or ‘strongly disagree’. Conditional logic was used so that only relevant questions were asked based on treatments used.

The five treatment categories defined in the patient survey were: hormonal therapies in tablet form (e.g., Primolut, contraceptive pill); LNG‐IUD (e.g., Mirena); other hormonal methods (e.g., Implanon, Depo Provera); surgery: EA/ER; and surgery: hysterectomy. Note, when considering EA/ER, the technique utilised by the regional single‐referral centre, is a first generation ER via transcervical resection of the endometrium with a hysteroscopic resectoscope.

The primary outcomes were to evaluate treatment efficacy and regret. Treatment effect was evaluated with the questions ‘my heavy periods were well controlled with this treatment’ and ‘if I had to do it over again, I would use this treatment again’ and regret evaluated with the question ‘I regret having used this treatment’. Secondary outcomes were to report on adverse effects, alignment of treatment with goals, and overall satisfaction with care.

### 2.4 Data Management

The data were imported from Qualtrics, and descriptive statistics were generated in Excel. A descriptive analysis is presented.

### 2.5 Ethics Approval

Ethical approval for this study was obtained from the Monash Health Human Research Ethics Committee, project number: 91012. Consent was obtained from participants via the survey.

## 3 Results

### 3.1 Response Rate

The survey was sent out to 1241 eligible participants, with a response received from 150 participants (response rate = 12%). Six Participants were excluded from the study as they selected ‘no’ when asked if they agreed to participate in the study. One further participant was excluded from the study on the basis of selecting ‘no’ when asked if they were over 18 years of age and had received care from a single referral centre for the management of heavy menstrual bleeding.

## 4 Discussion

### 4.1 Heavy Menstrual Bleeding Management

Most of the survey respondents were seen by a specialist Gynaecologist less than 2 months after being referred from a primary care physician (Table 1). There is a lack of current research pertaining to wait times to see a Gynaecologist in Victoria, Australia or more broadly. One Australian‐based study by Wood et al. [23] notes the detrimental effects that prolonged wait times to access specialist services may have on patients' physical and mental health.

**TABLE 1:** Heavy menstrual bleeding management.

	*n* (%)
**Wait time to see clinician once referred**	
< 2 weeks	26 (18.2%)
2 weeks–2 months	94 (65.7%)
2 months–6 months	19 (13.8%)
> 6 months	2 (1.4%)
Total respondents	141 (98.6%)
**Point of care ultrasound performed by clinician**	
Yes	120 (83.9%)
No	16 (11.2%)
Total respondents	136 (95%)

	*n* (% of total respondents)
**Treatments trialled in addition to hysterectomy**	
Hysterectomy + non‐surgical^a^ treatment	23 (16.1%)
Hysterectomy + EA/ER	2 (1.4%)
Hysterectomy + non‐surgical^a^ treatment + EA/ER	23 (16.1%)
Hysterectomy alone	19 (13.3%)
**Duration of heavy periods prior to hysterectomy**	
< 12 months	2 (3.0%)
1–3 years	12 (17.9%)
3–5 years	12 (17.9%)
5–10 years	14 (20.9%)
10+ years	25 (37.3%)
Did not respond	2 (3.0%)
Total number of respondents	65 (97.0%)
**Hysterectomy as an isolated treatment and duration of HMB**	
< 12 months	1 (5.3%)
1–3 years	6 (31.6%)
3–5 years	4 (21.1%)
5–10 years	6 (31.6%)
10+ years	2 (10.5%)
Total	19 (100%)

^a^ Combined hormonal, LNG‐IUD (Mirena), other hormones (Implanon, DepoProvera).

The majority of respondents had a point‐of‐care ultrasound (POCUS) performed by their clinician at the regional single‐referral centre to investigate their HMB (Table 1), which is especially important when considering a rural/regional patient cohort with potential limitations to accessing such services as a result of geographical barriers [24]. The availability of ultrasound services during specialist women's health consultations provides an efficient and effective care pathway, allowing for timely diagnosis and the facilitation of targeted discussions regarding treatment plans and planning for surgical interventions when required, within a singular appointment—thus reducing additional wait times for external imaging services, reducing financial burden on the patient, and reducing the need for subsequent follow‐up appointments with the Gynaecologist to review imaging reports from external service providers. According to Doig et al. [24], women who reside in regional and rural geographical locations are less likely to have access to POCUS during specialist Gynaecology appointments than their urban counterparts, highlighting the regional single‐referral centre of this study as an outlier in the provision of regional diagnostic services. All POCUS scans performed at the regional single‐referral centre were transvaginal (TV). Data was not collected regarding how many women had a formal ultrasound scan pre‐hysterectomy, nor regarding how many women had pelvic pain as a symptom of HMB.

Many of the respondents had already trialled other forms (both surgical and non‐surgical) of managing their HMB prior to electing to have a hysterectomy (Table 2). Some respondents indicated they had undergone a hysterectomy in the absence of trialling other treatments for HMB (Table 1). Of those who had hysterectomy alone, the majority had been living with HMB and associated symptoms for over 3 years (Table 1). As previously discussed, numerous studies [2, 3, 4, 5] emphasise the debilitating nature of HMB and it's associated symptoms on the person experiencing it, and the negative impact it may have on their quality of life across multiple domains, further highlighting the need to offer women experiencing HMB the most effective treatment possible in a timely manner, in order for them to be able to have the best possible quality of life.

**TABLE 2:** Treatment type.

	Used treatment, *n* (%)	Did not use treatment, *n* (%)	Unsure, *n* (%)	Total respondents, *n* (%)
**Treatment received for HMB**				
Oral hormones	72 (54.1%)	59 (44.4%)	2 (1.5%)	133 (93.0%)
Non‐oral hormones	24 (17.8%)	110 (81.5%)	1 (0.7%)	135 (94.4%)
LNG‐IUD	64 (47.1%)	72 (53.0%)	0 (0%)	136 (95.1%)
Endometrial ablation/resection	65 (48.1%)	62 (46.0%)	8 (5.9%)	135 (94.4%)
Hysterectomy	67 (50.0%)	66 (49.2%)	1 (0.7%)	134 (93.7%)

### 4.2 Efficacy and Regret of Treatment

The hysterectomy subgroup demonstrated the highest rates of satisfaction regarding control of HMB (Table 3), minimal adverse effects resulting from treatment (Table 3), and least treatment regret (Table 3). Whilst under 10% of respondents indicated they regret having had a hysterectomy (Table 3), it is important to consider why this may have been the case. This study did not ask respondents to cite the reasons for their regret; however, a study by Reddington et al. [25] with a similar reported rate of regret following hysterectomy noted predisposing factors to regret were a desire for future pregnancy or high levels of depression, anxiety or stress. Neither age nor parity were found to be associated with regret following hysterectomy [25]. As can be seen in Table 3, only 65% of respondents indicated their HMB was well‐controlled with EA/ER, which is an unexpected finding based on previous research [13] which demonstrated a greater degree of resolution of HMB over a one year follow‐up period after receipt of this treatment. It should be noted that 90.8% of respondents to the survey in this study who had received EA/ER supplied a response to the question regarding control of symptoms, which was lower than the response rate to this question for other treatment modalities. There is an imperative need for equitable utilisation of health resources, taking into consideration costs of theatre, need for time off work for recovery, alternative and less‐invasive methods that may provide adequate control of symptoms, such as the LNG‐IUD—it is important to note that the population of this study was presenting to a secondary specialist centre and is therefore likely to be over representative of those who have declined or experienced adversity from primary care medical and non‐surgical management. Despite this, 50% of respondents indicated satisfaction with LNG‐IUD, and 48.4% indicated it was effective in controlling their HMB.

**TABLE 3:** Efficacy and regret of treatment.

A. HMB well‐controlled with treatment							
B. Would choose treatment again							
C. Regret using treatment							
D. Suffered intolerable side‐effects							
Unless specified, percentages given for *n* refer to respondents to specific question		Strongly disagree, *n* (%)	Somewhat disagree, *n* (%)	Neutral, *n* (%)	Somewhat agree, *n* (%)	Strongly agree, *n* (%)	Total, *n* (% of total respondents)
Oral hormones	A.	23 (32.4%)	14 (19.7%)	6 (8.5%)	21 (29.6%)	7 (9.9%)	71 (98.6%)
	B.	31 (43.7%)	14 (19.7%)	7 (9.9%)	11 (15.5%)	8 (11.3%)	71 (98.6%)
	C.	27 (37.5%)	14 (19.4%)	18 (25.0%)	7 (9.7%)	6 (8.3%)	72 (100%)
	D.	14 (19.4%)	10 (13.9%)	14 (19.4%)	16 (22.2%)	18 (25%)	72 (100%)
Non‐oral hormones	A.	5 (20.8%)	4 (16.7%)	0 (0%)	8 (33.3%)	7 (29.2%)	24 (100%)
	B.	31 (43.7%)	14 (19.7%)	7 (9.9%)	11 (15.5%)	8 (11.3%)	71 (98.6%)
	C.	8 (33.3%)	2 (8.3%)	4 (16.7%)	6 (25.0%)	4 (16.7%)	24 (100%)
	D.	6 (25%)	1 (4.2%)	2 (8.3%)	10 (41.7%)	5 (20.8%)	24 (100%)
LNG‐IUD	A.	20 (32.3%)	6 (9.7%)	6 (9.7%)	9 (14.5%)	21 (33.9%)	62 (96.9%)
	B.	24 (38.7%)	3 (4.8%)	4 (6.5%)	7 (11.3%)	24 (38.7%)	62 (96.9%)
	C.	27 (43.5%)	6 (9.7%)	12 (19.4%)	3 (4.8%)	14 (22.6%)	62 (96.9%)
	D.	17 (27.4%)	12 (19.4%)	7 (11.3%)	6 (9.7%)	20 (32.3%)	62 (96.9%)
EA/ER	A.	5 (8.5%)	9 (15.3%)	7 (11.9%)	13 (22.0%)	25 (42.4%)	59 (90.8%)
	B.	4 (7.5%)	2 (3.8%)	2 (3.8%)	12 (22.6%)	33 (62.3%)	53 (81.5%)
	C.	40 (67.8%)	7 (11.9%)	5 (8.5%)	4 (6.8%)	3 (5.1%)	59 (90.8%)
	D.	33 (55.9%)	16 (27.1%)	3 (5.1%)	4 (6.8%)	3 (5.1%)	59 (90.8%)
Hysterectomy	A.	0 (0%)	1 (1.5%)	1 (1.5%)	2 (3.1%)	61 (93.8%)	65 (97.0%)
	B.	1 (1.6%)	3 (4.9%)	0 (0%)	4 (6.6%)	53 (86.9%)	61 (91.0%)
	C.	56 (86.2%)	4 (6.2%)	0 (0%)	2 (3.1%)	3 (4.6%)	65 (97.0%)
	D.	50 (76.9%)	5 (7.7%)	4 (6.2%)	4 (6.2%)	2 (3.1%)	65 (97.0%)

### 4.3 Goals of Care

Over 90% of respondents indicated they were satisfied that their concerns were heard by their clinician, and that the treatment recommendations were consistent with their goals and needs (Table 4). Regular evaluation of healthcare practices and receiving patient feedback is crucial to ensure delivery of evidence‐based, patient‐focused care. This study, prompted by Safer Care Victoria's ‘Best Care for Heavy Menstrual Bleeding’ enquiry [26], indicates that not only were the vast majority of respondents satisfied with the outcome of their hysterectomy (Table 3), this study also indicates that other treatments which patients had previously tried had not provided effective control of symptoms, and had a higher incidence of adverse effects and treatment regret when compared to hysterectomy (Table 3).

**TABLE 4:** Goals of care.

	*n* (%)
**Satisfaction that concerns heard by clinician**	
Extremely satisfied	107 (81.1%)
Somewhat satisfied	16 (12.1%)
Neutral	4 (3.0%)
Somewhat dissatisfied	3 (2.3%)
Extremely dissatisfied	2 (1.5%)
Total	132 (92.3% of survey respondents)
**Treatment recommendations consistent with goals and needs**	
Not at all consistent	1 (0.7%)
A little consistent	5 (3.8%)
Somewhat consistent	10 (7.6%)
Mostly consistent	27 (20.5%)
Very consistent	89 (67.4%)
Total	132 (92.3% of survey respondents)

Delivery of modern medical care involves a shared decision‐making process between doctor and patient, empowering patients to choose treatments that best align with their circumstances and health needs [27]. The concept of ‘too many’ hysterectomies warrants reconsideration, especially as it is generally a laparoscopic procedure with short post‐operative hospital stays, relatively short recovery period [28], minimal risk of complications, and, as supported by the findings of this study, is an effective and well‐tolerated treatment of HMB. Limiting access to this treatment to informed patients not only affects quality of life, but also imposes broader economic burdens such as lost productivity and the cost of provision of ineffective or non‐preferred treatment. For example, a woman experiencing HMB for 10 years could be absent from the workplace for up to 360 days, compared to a recovery period of two to six weeks post hysterectomy [28]. Hysterectomy remains a highly effective option for patients without future plans for pregnancy [18]. The availability of hysterectomy should be guided by informed, best‐practice care and should not be restricted by arbitrary geographical ‘targets’.

### 4.4 Limitations and Biases

This study's retrospective design and single‐centre focus may limit generalisability. Anonymous online surveys were sent out independently to episodes of care to reduce the risk of collection bias. Selection bias and recall bias may also affect the findings. The low response rate of 12% reflects the number of participants who completed the survey as a percentage of how many participants were sent a survey link. Confirmation of receipt of the survey was not undertaken, and as such may have yielded a lower response rate than the actual. As previously discussed, the study population was presenting to a secondary specialist centre and is therefore likely to be over‐representative of those who have declined or experienced adversity from primary care medical and non‐surgical management. Aside from confirming participants were over 18 years of age and had received treatment at the regional single referral centre, demographic detail was not collected, which may further impact generalisability and interpretability. Given the use of descriptive analysis, the results of this study are based upon historical findings [29], as such, caution should be exercised in using these results to predict future outcomes. Future research should investigate patient motivations and the impact of wait times on treatment choices, as well as directly comparing the various treatment options regarding the costs of absenteeism from the workplace, theatre costs, and recovery time.

## Conflicts of Interest

Dr. R. Dalton is a director of Obstetrics and Gynaecology Ballarat and owner of Ballart IVF. Dr. Ward received a Monash Health Foundation grant in 2021 and Epworth Medical Foundation grant in 2024. Dr. R. Dalton and Dr. Ward received an Australasian Gynaecological Endoscopy and Surgery (AGES) Research Grant in 2022 and 2024, as well as an Epworth Medical Foundation Capacity Building Grant 2023. No grants are relevant to this study.

## Data Availability

The data that support the findings of this study are available on request from the corresponding author. The data are not publicly available due to privacy or ethical restrictions.
